# Concurrent intracranial and spinal subdural hematomas: postoperative intracranial progression following surgical evacuation of spinal subdural hematoma: A case report and literature review

**DOI:** 10.1097/MD.0000000000045564

**Published:** 2025-10-31

**Authors:** Zehua Gong, Zhaohui Zhao

**Affiliations:** a Department of Neurosurgery, First Affiliated Hospital of Huzhou University, Huzhou, Zhejiang, China; b Department of Neurosurgery, The First People’s Hospital of Huzhou, Huzhou, Zhejiang, China.

**Keywords:** chronic subdural hematoma, laminectomy, spinal subdural hematoma, subdural hematoma

## Abstract

**Rationale:**

The co-occurrence of chronic subdural hematoma (CSDH) and spinal subdural hematoma (SSDH) is exceptionally rare, with ambiguous pathogenesis complicating management. This case aims to enhance understanding of its clinical trajectory, particularly the risk of intracranial progression after spinal surgery, which is critical for optimizing patient outcomes.

**Patient concerns:**

A 45-year-old woman presented with 3 days of severe lumbocrural pain and a 3-week history of headache after head trauma. She also reported 5 days of bowel dysfunction.

**Diagnoses:**

Lumbar magnetic resonance imaging revealed a lumbosacral SSDH (L2-S1). Cranial magnetic resonance imaging showed a right CSDH. Both hematomas were T1-isointense and T2-hyperintense without significant midline shift initially.

**Interventions:**

Oral atorvastatin (40 mg/day) was initiated for the CSDH. Emergency L4 hemilaminectomy for SSDH evacuation was performed due to intolerable pain and bowel dysfunction. On postoperative day 2, cranial CT showed CSDH progression with increased midline shift, prompting emergency burr-hole drainage.

**Outcomes:**

Lumbocrural pain resolved immediately postspinal surgery (visual analog scale: 9 to 2). Headache improved significantly postcranial drainage (numerical rating scale: 8 to 3). Bowel function normalized by discharge.

**Lessons:**

This case highlights that: SSDH can present with bowel dysfunction, a novel finding; postoperative intracranial hematoma progression is a real risk, necessitating vigilant neuroimaging surveillance after spinal evacuation; and the symptom sequence (cephalalgia preceding lumbalgia) supports the hematoma migration theory.

## 1. Introduction

Chronic subdural hematoma (CSDH) is a well-recognized pathological condition that frequently causes significant neurological deficits.^[1]^ Conversely, spinal subdural hematoma (SSDH) is an uncommon yet critical clinical occurrence whose pathogenesis remains poorly understood, thereby complicating its clinical management.^[2,3]^ The simultaneous occurrence of CSDH and SSDH constitutes an exceptionally rare phenomenon, with limited documented cases describing this combined presentation. Various hypothetical mechanisms have been proposed to elucidate the etiological basis for the concurrent development of intracranial and SSDH, encompassing the potential migration of hematoma from the cranial cavity to the spinal compartment or the coexistence of these hematomas secondary to shared predisposing risk factors.^[4]^

This report describes an exceptional case of concurrent presentation of CSDH and SSDH in a middle-aged female. We aimed to review the extant literature regarding the co-occurrence of these 2 pathological entities while examining the clinical characteristics, underlying mechanisms, and therapeutic strategies pertinent to this unique case. By presenting this case, we endeavored to enhance clinical awareness regarding the concurrent identification and management of CSDH and SSDH, with the ultimate objective of optimizing patient outcomes in this complex clinical scenario.

## 2. Case

**Figure 1.**
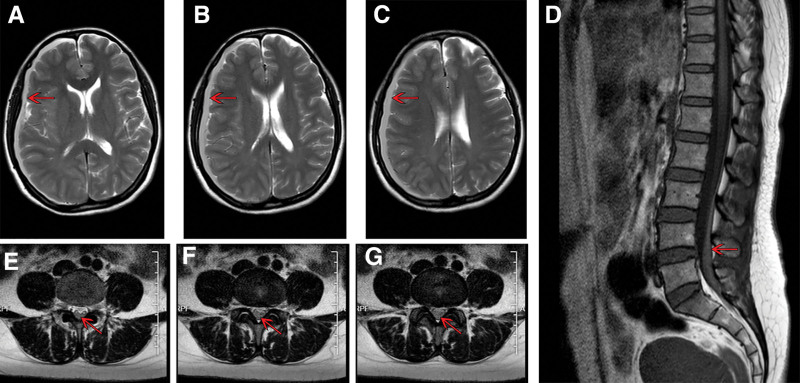
Lumbar spine and cranial MRI of the patient obtained on the day of admission. (A–C) Chronic subdural hematoma. (D–G) spinal subdural hematoma. The red arrow indicates a hematoma. MRI = magnetic resonance imaging.

A 45-year-old middle-aged woman presented to our hospital with a 3-day history of lumbocrural pain and 3-week history of headache. The medical history revealed that the patient had fallen from an electric bicycle 3 weeks prior, sustaining head trauma against the asphalt pavement without loss of consciousness. Although experienced a post-traumatic headache, she continued to work without seeking medical attention. 3 days before admission, she developed lower back pain radiating to the right thigh, which she reported was more severe than her persistent headache. On admission, the patient was alert, with a visual analog scale pain score of 9 for lumbocrural pain and a numerical rating scale pain score of 4 for her headache. Physical examination revealed a positive right straight-leg raise test without limb numbness or weakness. There had no history of anticoagulant therapy or coagulopathy. Magnetic resonance imaging (MRI) of the lumbar spine revealed an elongated fusiform intraspinal lesion at the lumbosacral level, isointense on T1-weighted imaging and slightly hyperintense on T2-weighted imaging, consistent with spinal subdural hematoma. Concurrent cranial MRI showed a linear subcranial lesion beneath the right calvarium, demonstrating similar signal characteristics (T1-isointense/T2-hyperintense), indicative of chronic subdural hematoma (Fig. 1). Elicited on further history taking, the patient described a 5-day cessation of defecation, representing acute deviation from her pre-traumatic bowel regularity. Physical examination revealed no abdominal signs, and both abdominal ultrasonography and abdominal CT scans yielded normal results.

**Figure 2.**
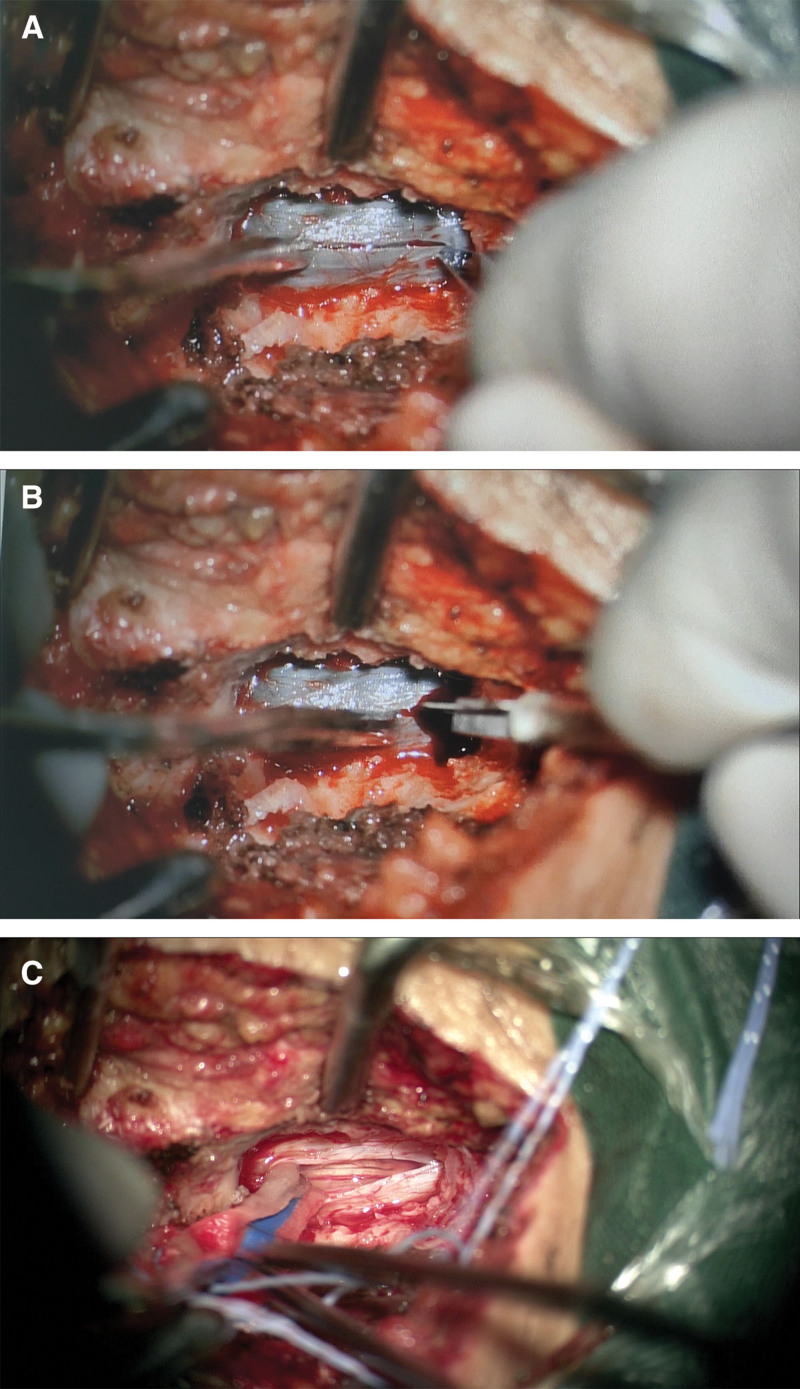
Intraoperative images. (A) Midline incision through the dura mater. (B) Upon opening the dura, dark red non-coagulated fluid (liquefied hematoma) actively drained from the subdural space. (C) Clear CSF was visualized beneath the arachnoid, and no evidence of subarachnoid hemorrhage was noted. CSF = cerebrospinal fluid.

The patient’s headache was tolerable, and given the small volume of the intracranial hematoma with no significant midline shift, oral atorvastatin calcium tablets (40 mg/day) were initially prescribed to promote subdural hematoma absorption. However, due to intolerable lumbocrural pain and the absence of bowel movements or urge for defecation for 5 days, surgical evacuation of the lumbar hematoma was performed on the second hospital day. A right L4 hemilaminectomy was performed, and no epidural hematoma was observed. The dura mater appeared darkened, tense, and non-pulsatile. A midline dural incision was made, while preserving the integrity of the underlying arachnoid layer. Upon opening the dura, dark red non-coagulated fluid (liquefied hematoma) actively drained from the subdural space. The spinal subdural hematoma was thoroughly irrigated until clear fluid was achieved (Fig. 2). The arachnoid layer remained intact, with no cerebrospinal fluid (CSF) leakage; clear CSF was visualized beneath the arachnoid, and no evidence of subarachnoid hemorrhage was noted. Postoperatively, lumbocrural pain resolved immediately (VAS score: 2). Postoperative lumbar MRI demonstrated adequate evacuation of the spinal subdural hematoma.

**Figure 3.**
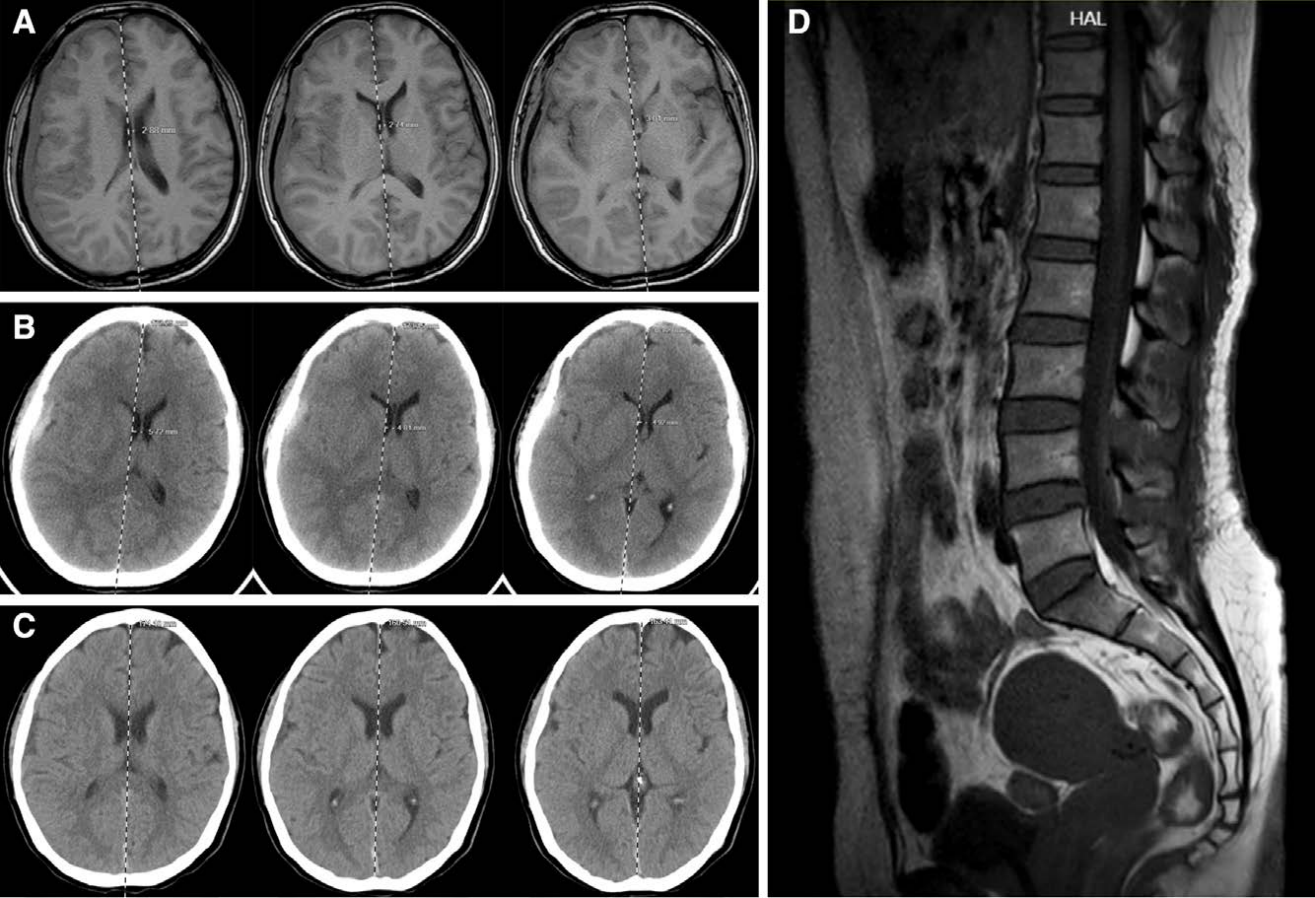
Postoperative imaging. (A) Admission MRI. (B) Postoperative Day 2 CT. (C) Postoperative CT. (D) Postoperative lumbar MRI. Compared to the Admission MRI, the Postoperative Day 2 CT demonstrated progressive midline shift. Postoperative CT demonstrated adequate evacuation of the hematoma with improvement in midline shift. Postoperative lumbar MRI demonstrated adequate evacuation of the spinal subdural hematoma. CT = computed tomography, MRI = magnetic resonance imaging.

On postoperative day 2, the patient experienced severe exacerbation of headache (NRS score: 8). Cranial CT revealed progression of the right chronic subdural hematoma with an increased midline shift compared to the initial MRI findings (Fig. 3). Emergency burr-hole drainage of the subdural hematoma was performed. By postoperative day 1, the headache had significantly improved (NRS score: 3) and follow-up cranial CT confirmed satisfactory hematoma evacuation (Fig. 3). At discharge, 2 weeks postoperatively, the patient regained normal bowel function with restored spontaneous defecation, consistent with her pre-traumatic pattern.

## 3. Discussion

The co-occurrence of chronic subdural hematoma (CSDH) and spinal subdural hematoma (SSDH) is an exceptionally rare clinical phenomenon, with only 33 documented cases of concomitant cranial and SSDH reported to date, including the present case (Table 1).^[4–32]^ To our knowledge, this represents a singular case report distinguished by 2 exceptional features: it is the first documented description of bowel dysfunction secondary to spinal subdural hematoma compression and the patient underwent 2 emergent surgeries during hospitalization due to rapid intracranial progression shortly after hemilaminectomy drainage, a clinical course scarcely reported in the existing literature.

**Table 1:** Summary of reported cases of coexistence of CSDH and SSDH.

Category	Case	Age(years)/Sex	Predisposing event	First lesion detected	Clinical Presentation	Dominant side of CSDH	Location of SSDH	Treatment		Prognosis
								Intracranial	Spinal	
Trauma-related	1^[4]^	67/F	Head trauma	Simultaneous	Bilateral leg weakness	Bilateral	L4-S1	Burr-hole drainage	Laminectomy	Good
	2^[5]^	23/F	Head trauma	Intracranial	Progressive bone pain, leg numbness	Unilateral	L4-S2	Conservative	Laminotomy	Good
	3^[6]^	58/M	Head trauma	Intracranial	Headache, leg pain, low back pain	Bilateral	T1-S1	Burr-hole drainage	Conservative	Good
	4^[7]^	71/M	Head trauma	Spinal	Bilateral leg weakness	Bilateral	L2-L5	Burr-hole drainage	Resection	Good
	5^[8]^	76/M	Head trauma	Intracranial	right hemiparesis	Unilateral	T11-S2	Burr-hole drainage	Hemilaminectomy	Good
	6^[9]^	56/M	Head trauma	Intracranial	L4-S1 radiculopathy	Hemispheric subdural	L1-S2	Conservative	Surgical decompression	Good
	7^[10]^	73/F	Head trauma	Intracranial	Altered consciousness	Bilateral	T4-T10	Conservative	Conservative	Good
	8^[11]^	47/M	Head trauma	Intracranial	Right hemiparesis, low back pain	Bilateral	L3-S1	Burr-hole drainage	Conservative	Good
	9^[12]^	47/F	Head trauma	Simultaneous	Acute headache, aggravated back pain	Tentorial	L5-S2	Conservative	Conservative	Good
	10^[13]^	62/M	Head trauma	Intracranial	Low back pain, weakness	Bilateral	L2-L5	Conservative	Conservative	Good
	11^[14]^	83/F	Head trauma	Simultaneous	Asymptomatic	Bilateral	L3-S1	Conservative	Laminotomy	Good
	12^[14]^	70/F	Head trauma	Simultaneous	Asymptomatic	Bilateral	L4-S2	Burr-hole drainage	Conservative	Good
	13^[15]^	15/M	Trauma	Simultaneous	Rigidity, nausea, leg pain	Hemispheric fissure	T11/12-L4	Conservative	Conservative	Good
	14^[16]^	57/F	Trauma	Spinal	Low back pain, leg pain	Bilateral	L5-S1	Burr-hole drainage	Laminotomy	Good
	15^[17]^	45/F	Head trauma	Spinal	Headache, low back pain, hemiparesis	Bilateral	L4-S3	Conservative	Hemilaminectomy	Good
	16^[18]^	40/M	Head trauma	Simultaneous	Low back pain	Bilateral	L2-S1	Burr-hole drainage	Conservative	Good
	17^[19]^	78/M	Trauma	Intracranial	Bilateral leg pain	Hemispheric fissure	L5-S2	Conservative	Conservative	Good
	18^[20]^	60/M	Head trauma	Intracranial	Headache, low back pain	Bilateral	L5-S2	Evacuation of chronic SDH	Conservative	Good
	Present case	45/F	Head trauma	Simultaneous	Headache, low back pain, bowel dysfunction	Unilateral	L2-S1	Burr-hole drainage	Hemilaminectomy	Good
Subtotal				19 cases		Mean: 56.5 yr			10M:9F	
Spontaneous	20^[19]^	80/M	Spontaneous	Simultaneous	Headache, low back pain, leg pain	Bilateral posterior fossa	L5-S2	Conservative	Imaging-guided drainage	Good
	21^[20]^	66/M	Spontaneous	Spinal	Bilateral leg pain, paralysis	Bilateral	L2-S1	Evacuation of chronic SDH	Conservative	Good
	22^[21]^	59/M	Spontaneous	Simultaneous	Low back pain, bilateral leg pain	Bilateral	T11-S1	Conservative	Conservative	Good
	23^[22]^	73/M	Spontaneous	Simultaneous	Bilateral sciatic pain	Bilateral	L3-S2	Craniotomy	Conservative	Good
	24^[23]^	39/F	Spontaneous	Spinal	Headache, low back pain, leg pain	Bilateral	L4-S2	Burr-hole drainage	Conservative	Good
	25^[24]^	82/F	Spontaneous	Intracranial	Lower limb numbness, weakness	Bilateral	T-L1	Burr-hole drainage	Conservative	Good
	26^[25]^	70/M	Spontaneous	Simultaneous	Low back pain, paraparesis	Bilateral	L4-S1	Burr-hole drainage	Conservative	Good
	27^[26]^	64/M	Spontaneous	Intracranial	Headache	Bilateral	L5-S	Conservative	Conservative	Good
Subtotal				8 cases		Mean: 66.6 yr			6M:2F	
Anticoagulation/Antiplatelet	28^[27]^	67/F	Antiplatelet therapy	Simultaneous	Headache, back pain, leg weakness	Bilateral	L4-S1	Burr-hole drainage	Conservative	Good
	29^[28]^	11/M	Aplastic Anemia	Intracranial	Neck/back pain, headache	Bilateral	C1-S	Conservative	Conservative	Good
	30^[29]^	56/F	Antiplatelet therapy	Spinal	Allergic reaction	Bilateral	T12-S1	Burr-hole drainage	Conservative	Good
	31^[30]^	77/M	Anticoagulation	Spinal	Back pain, leg weakness, coma	Bilateral	L4-S5	Burr-hole drainage	Conservative	Good
Subtotal				4 cases		Mean: 52.8 yr			2M:2F	
Other	32^[31]^	12/M	Undifferentiated	Simultaneous	Headache, low back pain	Bilateral posterior fossa	C1-S3	Conservative	Conservative	Good
	33^[32]^	78/F	Iatrogenic injury	Intracranial	Headache, bilateral leg weakness	Posterior fossa	S1-S2	Conservative	Conservative	Good
Subtotal				2 cases		Mean: 45 yr			1M:1F	

CSDH = chronic subdural hematoma, SSDH = spinal subdural hematoma.

Despite these well-characterized clinical observations, the precise mechanisms underlying their simultaneous occurrence remain unclear. Several theoretical frameworks have been proposed to achieve this purpose. One hypothesis posits the potential migration of the CSDH to the spinal compartment, suggesting that cranial SDH may undergo caudally directed redistribution along the subdural space.^[29,30,33]^ Ultrastructural investigations have revealed that cranial and spinal subdural compartments are anatomically discontinuous under normal physiological conditions and are populated by neuroepithelial cells. The mechanical disruption of these neuroepithelial barriers may establish communication between the intracranial and spinal subdural spaces. Subdural fluid accumulation can induce structural compromise of neuroepithelial cells, generating fissures at the craniospinal interface that permit SDH redistribution from intracranial to spinal regions.^[34]^ In the current case, the patient developed a headache following head trauma, followed by lumbodorsal pain. Based on the temporal sequence of symptom presentation, it is reasonable to postulate that the patient initially developed CSDH, followed by SSDH. This chronological pattern aligns with a case reported by Bortolotti et al, involving a 23-year-old female who presented with progressive lower back pain and sciatica 4 days after a traumatic cranial SDH.^[5]^ MRI revealed a spinal subdural hematoma extending from the thoracic spine to the lumbar spine (T12-L1). Surgical intervention via laminectomy confirmed the subdural origin of the hematoma, with intact arachnoid membranes and no evidence of subarachnoid hemorrhage after evacuation. These findings strongly support the hypothesis that caudal migration of the intracranial subdural blood along the spinal canal is the underlying mechanism. Similarly, Matsumoto et al documented a 58-year-old male who developed SSDH 3 days after surgical evacuation of a CSDH secondary to head trauma, postulating gravitational migration of liquefied hematoma from the cranial to the dependent spinal subdural compartments as the pathogenic mechanism.^[6]^

Furthermore, SSDH evacuation may precipitate intracranial SDH development or the progression of preexisting hematomas. In the current case, the patient experienced marked exacerbation of cephalalgia on postoperative day 2 following lumbar hematoma evacuation, with cranial CT demonstrating SDH progression evidenced by an increased midline shift compared to baseline MRI findings. This clinical trajectory finds corroboration in existing literature: Hajare et al reported a 71-year-old male presenting with bilateral lower extremity pain and gait disturbance (devoid of intracranial hypertension manifestations such as headache or vomiting) who developed acute hemiparesis and sensory deficits following L2-L5 SSDH evacuation, with subsequent cranial CT revealing de novo SDH.^[7]^ Similar cases have been reported in which symptomatic intracranial SDH manifested within 4 days following traumatic spinal SDH.^[29]^ Notably, Uto et al described a 77-year-old male with an 11-day history of lumbosacral pain accompanied by lower extremity radiculopathy and right foot dysesthesia, whose SSH extending from L4 to S1, followed by tinnitus and visual illusions 30 days later, with cranial CT confirming chronic SDH with midline displacement.^[35]^ Therefore, the emergence of intracranial hypertension symptoms (cephalalgia, nausea, and emesis) in SSDH patients warrants prompt neuroimaging evaluation to exclude de novo intracranial SDH formation or progression of preexisting lesions.

An alternative theoretical framework posits that SSDH formation may directly correlate with trauma to the lumbosacral region, analogous to the mechanistic basis of CSDH development after cranial trauma. Biomechanical studies suggest that concurrent impact to the cephalic and spinal regions, even from low-energy trauma, may induce simultaneous development of intracranial and spinal SDH.^[36–40]^ Nevertheless, given the paucity of isolated spinal SDH case reports,^[41]^ definitive diagnosis of such co-occurrence often requires intraoperative confirmation of membrane continuity, as conventional neuroimaging modalities demonstrate limited discriminative capacity. Surgical and histopathological verification of concomitant intracranial-spinal CSDH has been established through operative observations by Leber et al and Tillich et al.^[42,43]^ This concept is further supported by Yoshiki Fujikawa clinicopathological analysis, which revealed striking histomorphological similarities between spinal SDH neomembranes and their intracranial CSDH counterparts, suggesting potential pathophysiological congruence.^[8]^

From a vascular perspective, spinal trauma may precipitate bridging vein disruption or dural vessel injuries. The spinal subdural compartment contains bridging vein analogs that exhibit structural homology with their intracranial counterparts, but demonstrate heightened fragility. These vulnerable vascular structures are susceptible to rupture following mechanical stress or iatrogenic intervention (e.g., lumbar puncture).^[44,45]^ Brandt^[46]^ seminal work provided clinical evidence of SSDH development after lumbar puncture, while recent anatomical investigations by Komiyama et al elucidated the critical role of radiculomedullary veins as spinal equivalents of bridging veins in hemorrhage pathogenesis.^[47]^ This mechanistic paradigm demonstrates remarkable concordance with Bullock vascular injury hypothesis for intracranial SDH formation, reinforcing the concept of unified pathomechanisms across neuraxial compartments.^[48]^

The third theoretical construct proposes distinct pathophysiological divergence between SSDH and CSDH.^[49]^ This hypothesis postulates that abrupt elevations in intra-abdominal pressure or intrathoracic pressure (ITP) induced by Valsalva maneuvers, such as paroxysmal coughing, emesis, defecation, or weightlifting, may precipitate acute pressure surges within the spinal segmental vasculature (e.g., lateral spinal arteries). The rigid anatomical constraints imposed by spinal osseoligamentous structures prevent cerebrospinal fluid (CSF) pressure equilibration, potentially culminating in rupture of the spinal subdural microvasculature and subsequent hematoma formation. Shimada et al provided direct clinicopathological substantiation through the documentation of a coexistent lumbar ligamentum flavum hematoma and SSDH, establishing a mechanistic link between abdominal pressure dysregulation and spinal vascular compromise.^[50]^ Earlier observations by Rader documented the development of spontaneous SSDH in trauma-free patients, supporting pressure-mediated vascular injury as an independent etiological pathway.^[49]^ Postmortem anatomical investigations further corroborate this mechanism, with Stone and Jones demonstrating the high sensitivity of spinal epidural venous plexuses to abdominal pressure fluctuations through cadaveric pressure-response analyses.^[51]^ Therefore, in patients without evident trauma but presenting with chronic intra-abdominal hypertension risk factors (e.g., COPD, chronic constipation, and obesity), the possibility of SSDH should be considered. During clinical management, surgical evacuation of the hematoma should be combined with active control of intra-abdominal hypertension risk factors (including antitussive measures for chronic cough and laxative measures for constipation) to mitigate recurrence risk.

An additional pathophysiological consideration involves potential transarachnoid extension of subarachnoid hemorrhage into the subdural compartment. This theoretical model postulates that mechanical disruption of the arachnoid membrane secondary to trauma, lumbar puncture, or vascular anomalies may permit displacement of subarachnoid blood into the subdural space. Early clinical evidence by Banach documented spontaneous spinal subarachnoid hemorrhage progression to SSDH, suggesting potential microscopic arachnoid defects serving as hematoma conduits.^[52]^ Furthermore, Rengachary and Szymanski demonstrated that arterial-origin subdural hematomas (e.g., vascular malformations) may induce secondary arachnoid injury through pulsatile vascular erosion.^[53]^ This pathophysiological continuum demonstrates parallels with its intracranial counterparts, as evidenced by Barton and Tudor observations of subdural extension following ruptured cerebral aneurysms.^[54]^ Nevertheless, this hypothesis currently faces 2 principal limitations: lack of direct histopathological evidence demonstrating arachnoid microperforations and potential mechanistic overlap with hematoma migration theories.

Contemporary research delineates 4 principal mechanistic paradigms underlying the co-occurrence of cranial and spinal subdural hematomas: the migration theory postulates cranial-to-spinal hematoma extension through disrupted neuroepithelial barriers; the common trauma theory implicates concurrent cephalospinal injury in dual hematoma formation; the independent occurrence theory emphasizes intra-abdominal pressure/ITP-induced vascular pressure surges as a unique spinal-specific mechanism; and the transarachnoid extension theory proposes subdural hematoma formation via subarachnoid hemorrhage transmigration.

The present case demonstrates temporal symptom evolution (cephalalgia preceding lumbalgia), absence of definitive neomembranes intraoperatively, and postoperative intracranial hematoma progression, features concordant with migration theory cases reported by Bortolotti et al and Hajare et al.^[5–7]^ Nevertheless, clinical management requires comprehensive consideration of the temporal relationships between traumatic exposures/pressure-inducing episodes and symptom chronology, combined with serial neuroimaging protocols to monitor hematoma dynamics and histopathological assessment, along with heightened awareness of potential intracranial hematoma progression following spinal surgical interventions. Notably, these mechanisms may operate synergistically or exhibit a case-specific predominance. It should be noted that this study has limitations, primarily its nature as a single-center case report. Future elucidation of the precise pathomechanisms demands multicenter case accrual complemented by advanced imaging-pathology correlation studies, ultimately informing evidence-based diagnostic and therapeutic algorithms. Nonetheless, the findings from this study provide valuable insights for the clinical understanding of this rare condition.

## Author contributions

**Writing – original draft:** Zehua Gong.

**Writing – review & editing:** Zhaohui Zhao.
